# Early prediction of acute kidney injury after therapeutic paracentesis in decompensated liver cirrhosis: diagnostic value of IL-18, KIM-1, and FGF-23

**DOI:** 10.1080/0886022X.2026.2644101

**Published:** 2026-03-17

**Authors:** Ahmed Fayed, Ahmed Ramadan, Tarek Ramzy, Amr Shaker

**Affiliations:** ^a^ Nephrology Unit, Internal Medicine Department, Kasr Alainy School of Medicine, Cairo University, Cairo, Egypt; ^b^ Endemic Medicine Department, Kasr Alainy School of Medicine, Cairo University, Cairo, Egypt; ^c^ Chemical Pathology Department, Kasr Alainy School of Medicine, Cairo University, Cairo, Egypt

**Keywords:** AKI, IL-18, KIM-1, FGF-23, paracentesis

## Abstract

Acute kidney injury (AKI) is a potentially fatal complication in patients with decompensated cirrhosis having therapeutic paracentesis, and early diagnosis is still difficult because of the low sensitivity of serum creatinine. We preemptively tested serum interleukin-18 (IL-18), kidney injury molecule-1 (KIM-1), and fibroblast growth factor-23 (FGF-23) in early prediction of post-paracentesis AKI in 80 hospitalized patients with tense ascites and normal baseline kidney function. Samples of blood were collected 2 h pre-paracentesis and 24 h post-paracentesis. Based on the Kidney Disease: Improving Global Outcomes definition, AKI, which happened within 72 h, was present in 40% of patients. Biomarkers levels at baseline were comparable. In 24 h of paracentesis, IL-18, KIM-1, and FGF-23 were high in patients who later developed AKI (all *p* value below .001) and had high-absolute changes than the non-AKI group. The largest discriminative performance was observed of FGF-23 (area under the curve 0.959), then IL-18 (0.931), and KIM-1 (0.903). In multivariate logistic regression controlling MELD-Na score, serum albumin, baseline eGFR and ascitic fluid volume, the post-paracentesis FGF-23 was still independently connected with AKI. Biomarkers of tubular injury, especially FGF-23, which increased shortly after the procedure were strongly correlated with future AKI and could increase risk stratification pending external validation.

## Introduction

The large-volume paracentesis that is done for therapeutic reasons is one of the most important treatments for tense ascites in patients with decompensated cirrhosis. Even after albumin replacement, paracentesis-induced circulatory dysfunction (PICD) remains a clinically significant complication; it is characterized by effective arterial hypovolemia, neurohormonal activation, and renal vasoconstriction. Such a shift in hemodynamics can lead to acute kidney injury (AKI) especially among those who have poor renal reserve [1].

It would be very difficult to identify early post-paracentesis AKI because an increase in serum creatinine happens later and might not fully reflect the extent of renal dysfunction in cirrhosis due to decreased muscle mass and changed creatinine kinetics. Therefore, biomarkers that indicate early tubular stress rather than late functional decline could possibly enhance risk stratification after paracentesis [2–4].

Traditional renal biomarkers are therefore not sufficient for the early detection of subtle or evolving kidney injury in cirrhotic patients. Thus, a lot of research has been done on finding new biomarkers that can predict AKI before serum creatinine can do it. Ideally, such biomarkers would reflect either true changes in glomerular filtration or early tubular stress and injury which are the initial stages of AKI pathogenesis. The incorporation of new biomarkers into AKI evaluation may enhance patient stratification, timely therapeutic decisions as well as drug development strategies. This could eventually lead to a decrease in morbidity and mortality related to AKI; however, proper validation together with integration into established clinical parameters and outcomes is still required. Most studied among interleukin-18 (IL-18), kidney injury molecule-1 (KIM-1), and fibroblast growth factor-23 (FGF-23) are these markers that increase significantly following ischemic or inflammatory tubular injury and might be indicative of renal stress before overt functional decline occurs [5–9]. Among this background, the current study assessed the clinical usefulness of serum tubular injury biomarkers IL-18, KIM-1, and FGF-23 in the early detection of AKI among patients with liver cirrhosis receiving therapeutic paracentesis.

## Materials and methods

This prospective observational cohort study was conducted at Kasr Al-Ainy Hospital, Cairo University, from October 2022 to October 2023. The study protocol received approval from the Research Ethics Committee of the Faculty of Medicine, Cairo University (Approval No. N-150-2023). All procedures were in accordance with the principles of the Declaration of Helsinki. Written informed consent was obtained from all participants prior to their enrollment in the study.

Eighty consecutive adult patients with decompensated liver cirrhosis and tense ascites requiring therapeutic paracentesis were recruited for the study. A control group of 20 apparently healthy age-matched individuals was also included to provide reference values for the biomarkers that are being studied. The diagnosis of liver cirrhosis was made based on clinical features, biochemical abnormalities, and radiological findings which revealed reduced liver size, portal vein dilatation and presence of ascites on abdominal ultrasonography.

Eligible patients were 18 years or older with decompensated cirrhosis requiring therapeutic paracentesis who had baseline renal function preserved (serum creatinine <1.5 mg/dL) and urine output adequate (>500 mL/day) before this procedure. Exclusion criteria included active or occult infection such as spontaneous bacterial peritonitis; hepatocellular carcinoma or other malignancies; active gastrointestinal bleeding; preexisting chronic kidney disease estimated glomerular filtration rate [eGFR] < 60 mL/min/1.73 m^2^); established acute kidney injury or end-stage renal disease at baseline; prior kidney transplantation; and congestive heart failure.

At baseline, all participants had a complete clinical and laboratory evaluation that included demographic data, body mass index, and mean arterial pressure. Renal function was assessed by serum creatinine measurement and eGFR was calculated using the Chronic Kidney Disease Epidemiology Collaboration (CKD-EPI) equation [10]. Severity of liver disease was assessed by the Model for End-Stage Liver Disease–Sodium (MELD-Na) score according to United Network for Organ Sharing (UNOS) recommendations with serum sodium values adjusted between 125–137 mmol/L [11].

Therapeutic paracentesis was performed under ultrasound guidance by experienced clinicians. Patients were monitored closely during the procedure with continuous vital sign checks for blood pressure, heart rate, and respiratory rate. According to institutional policies and practices, the amount of deducted fluid was generally maintained at 5 L for each session so as to minimize percutaneous CT-induced circulatory failure. When indicated by clinical status, residual ascites was left and repeat procedures performed as required. To minimize the risk of paracentesis-induced circulatory dysfunction, intravenous human albumin at a dose of 20 g was given after removing 3–5 liters of ascitic fluid, per standard clinical practice.

Patients were followed up prospectively for acute kidney injury after paracentesis. AKI was defined based on the Kidney Disease: Improving Global Outcomes criteria as an increase in serum creatinine by at least 0.3 mg/dL within 48 h or an increase to 1.5 times or more from baseline within seven days or reduction in urine output to less than 0.5 mL/kg/h for 6 h [12]. On this basis, patients were classified into AKI and non-AKI groups for further analysis.

Venous blood samples were collected at two time points: (1) immediately before paracentesis (within 6 h prior to the procedure) and (2) within 24 h after paracentesis. This allowed assessment of both baseline levels and early post-procedural changes in serum biomarkers. Serum levels of IL-18, KIM-1, and FGF-23 were assessed using enzyme-linked immunosorbent assay kits from a commercial source (Shanghai Sunred Biological Technology Co., Ltd., China) based on the manufacturer’s protocol. All biomarker assays were performed by laboratory personnel blinded to clinical data and AKI outcomes.

### Statistical analysis

The statistical analyses were performed using IBM SPSS Statistics version 26 software (IBM Corp., Armonk, NY, USA). A post-hoc power analysis was performed using the difference in post-paracentesis FGF-23 levels between AKI vs non-AKI. With 32 cases of AKI in 80 patients, the study has achieved >80% power to detect a large effect size (Cohen’s *d* > 0.8) at a two sided-α level of 0.05. Continuous variables were expressed as mean ± standard deviation or median with interquartile range where appropriate; categorical variables were expressed in terms of frequencies and percentages. Comparisons between AKI and non-AKI groups were made by the Mann–Whitney U test for non-normally distributed continuous variables and by Chi-square or Fisher’s exact test for categorical variables [13,14]. Renal biomarkers and parameters of kidney function were related by Spearman’s rank correlation coefficient. The diagnostic performance of IL-18, KIM-1, and FGF-23 in predicting post-paracentesis AKI was assessed through receiver operating characteristic curve analysis with corresponding area under the curve values calculated along with optimal cutoff values determined. A two-tailed *p* value of <.05 was considered statistically significant. Multivariable logistic regression was performed to evaluate independent predictors of post-paracentesis AKI. Variables with *p* < .10 in univariable analysis and clinically relevant factors (MELD-Na, baseline serum creatinine, and ascitic fluid volume removed) were included in the final model.

## Results

### Baseline characteristics

A total of 80 patients with decompensated liver cirrhosis that were being paracentesized therapeutically were used. The average age was 54.310.9 years and 36 (45) males. The mean MELD score was 18.7 ± 9.1. Baseline serum creatinine was 0.88 ± 0.15 mg/dL and all the patients maintained good renal functioning at the time of enrollment. The median amount of removed ascitic fluid was 5.0 L (interquartile range [IQR] 4.55). Every patient was fed on intravenous albumin replacement. None of the patients needed vasopressor during paracentesis (Table 1).

**Table 1.** Baseline demographic and laboratory characteristics according to development of AKI.

Variable	AKI (*n* = 32)	Non-AKI (*n* = 48)	*p* Value
Age (years), mean ± *SD*	55.6 ± 10.7	53.1 ± 11.1	.64ᵃ
Male sex, *n* (%)	16 (50.0)	22 (45.8)	.74ᵇ
MELD score, mean ± *SD*	19.2 ± 8.5	18.4 ± 9.5	.34ᵃ
Hemoglobin (g/dL), mean ± *SD*	9.2 ± 1.3	9.8 ± 0.9	.25ᵃ
Platelet count (×10³/µL), mean ± *SD*	125 ± 12.3	120 ± 11.3	.83ᵃ
INR, mean ± *SD*	1.8 ± 0.4	1.9 ± 0.5	.48ᵃ
Serum albumin (g/dL), mean ± *SD*	2.45 ± 0.5	2.50 ± 0.6	.39ᵃ
Baseline serum creatinine (mg/dL), mean ± *SD*	0.89 ± 0.17	0.87 ± 0.14	.57ᵃ
eGFR (mL/min/1.73 m²), mean ± *SD*	91.4 ± 18.6	94.2 ± 16.9	.41ᵃ
CRP (mg/dL), median (IQR)	17.5 (15.2–19.8)	14.9 (13.1–17.3)	.07ᶜ
Paracentesis volume (L), median (IQR)	5.4 (5.0–5.8)	5.2 (4.5–5.5)	.79ᶜ
KIM-1 (U/L), median (IQR)	0.72 (0.61–0.85)	0.69 (0.58–0.80)	.41ᶜ
IL-18 (U/L), median (IQR)	3.4 (2.8–3.9)	3.2 (2.7–3.8)	.52ᶜ
FGF-23 (pg/mL), median (IQR)	29 (27–33)	28 (26–32)	.48ᶜ

*Notes:* Values are presented as mean ± *SD* for normally distributed variables and median (IQR) for non-normally distributed variables. Estimated glomerular filtration rate (eGFR) was calculated using the CKD-EPI equation. MELD: Model for End-Stage Liver Disease; INR: international normalized ratio; CRP: C-reactive protein; IQR: interquartile range; eGFR: estimated glomerular filtration rate; CKD-EPI: Chronic Kidney Disease Epidemiology Collaboration.

ᵃStudent’s *t* test.

ᵇChi-square test.

ᶜMann–Whitney *U* test. AKI was defined according to KDIGO criteria within 72 h after paracentesis.

No significant difference in baseline demographic and laboratory parameter was detected between patients who later developed AKI or those that did not (Table 2).

**Table 2.** Clinical and laboratory characteristics according to development of acute kidney injury (AKI).

Variable	AKI (*n* = 32)	Non-AKI (*n* = 48)	*p* Value
Age (years), mean ± *SD*	55.6 ± 10.7	53.1 ± 11.1	.64
Male sex, *n* (%)	16 (50)	22 (45.8)	.74
MELD score, mean ± *SD*	19.2 ± 8.5	18.4 ± 9.5	.34
Hemoglobin (g/dL), mean ± *SD*	9.2 ± 1.3	9.8 ± 0.9	.25
Platelets (×10³/µL), mean ± *SD*	125 ± 12.3	120 ± 11.3	.83
INR, mean ± *SD*	1.8 ± 0.4	1.9 ± 0.5	.48
Serum albumin (g/dL), mean ± *SD*	2.45 ± 0.5	2.50 ± 0.6	.39
Baseline creatinine (mg/dL), mean ± *SD*	0.89 ± 0.17	0.87 ± 0.14	.57
CRP (mg/dL), mean ± *SD*	17.5 ± 3.5	14.9 ± 3.2	.71
Paracentesis volume (L), median (IQR)	5.4 (5.0–5.8)	5.2 (4.5–5.5)	.79
Post-paracentesis IL-18 (U/L), median (IQR)	9.1 (7.2–11.4)	3.0 (2.7–3.5)	<.001
Post-paracentesis KIM-1 (U/L), median (IQR)	2.0 (1.5–2.6)	0.6 (0.5–0.8)	<.001
Post-paracentesis FGF-23 (pg/mL), median (IQR)	178 (142–224)	30 (28–33)	<.001

*Note:* MELD: Model for End-Stage Liver Disease; INR: international normalized ratio; CRP: C-reactive protein; IL-18: interleukin-18; KIM-1: kidney injury molecule-1; FGF-23: fibroblast growth factor-23; IQR: interquartile range.

### Incidence of AKI

KDIGO criteria of serum creatinine showed that AKI is seen in 32/80 (40%); in 48–72 h post-paracentesis. None of the patients were able to meet the urine output criteria independently of their creatinine elevation.

### Biomarker levels, correlation, diagnostic performance, and regression analysis

There were no significant differences in pre-paracentesis serum IL-18, KIM-1, and FGF-23 in the groups of patients who were later found to have AKI and those who did not (all *p* > .05). Conversely, all three biomarkers were highly increased in the AKI group within 24 h of paracentesis. The median IL-18 of patients with AKI was 9.1 U/L (IQR 7.211.4) and 3.0 U/L (IQR 2.73.5) with AKI in patients who did not develop the illness (*p* < .001). The median KIM-1 was 2.0 U/L (IQR 1.52.6) and 0.6 U/L (IQR 0.50.8), respectively (*p* < .001). On the same note, median FGF-23 levels in AKI group were significantly higher, at 178 pg/mL (IQR 142,224), than in non-AKI group, which was also 30 pg/mL (IQR 2833) (*p* < .001). Patients who had no AKI showed no clinically meaningful change in the biomarker concentrations following the procedure (Table 2).

**Figure 1.**
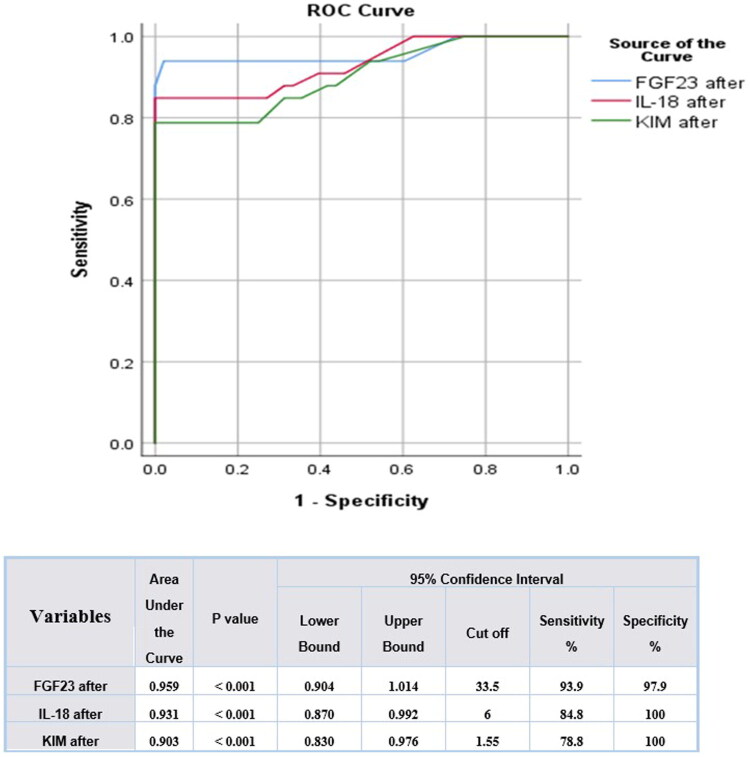
ROC curve for detection of best cutoff value of tubular injury markers for detection of AKI in patients with chronic liver disease who undergo paracentesis. IL-18: interleukin-18; KIM-1: kidney injury molecule-1; FGF-23: fibroblast growth factor-23.

All three biomarkers showed high discrimination using receiver operating characteristic (ROC) analysis. FGF-23 was the highest area under the curve (AUC 0.959; 95% CI 0.91 0.99) followed by IL-18 (AUC 0.931; 95% CI 0.87 0.98) and KIM-1 (AUC 0.903; 95% CI 0.83 0.96). The best cutoff point was FGF-23 (33.5 pg/mL) with the sensitivity of 93.9 and specificity of 97.9. In the case of IL-18, the sensitivity and specificity of 6 U/L was 84.8 and 100, respectively, and sensitivity and specificity of 1.55 U/L was 78.8 and 100, respectively (Figure 1).

**Figure 2.**
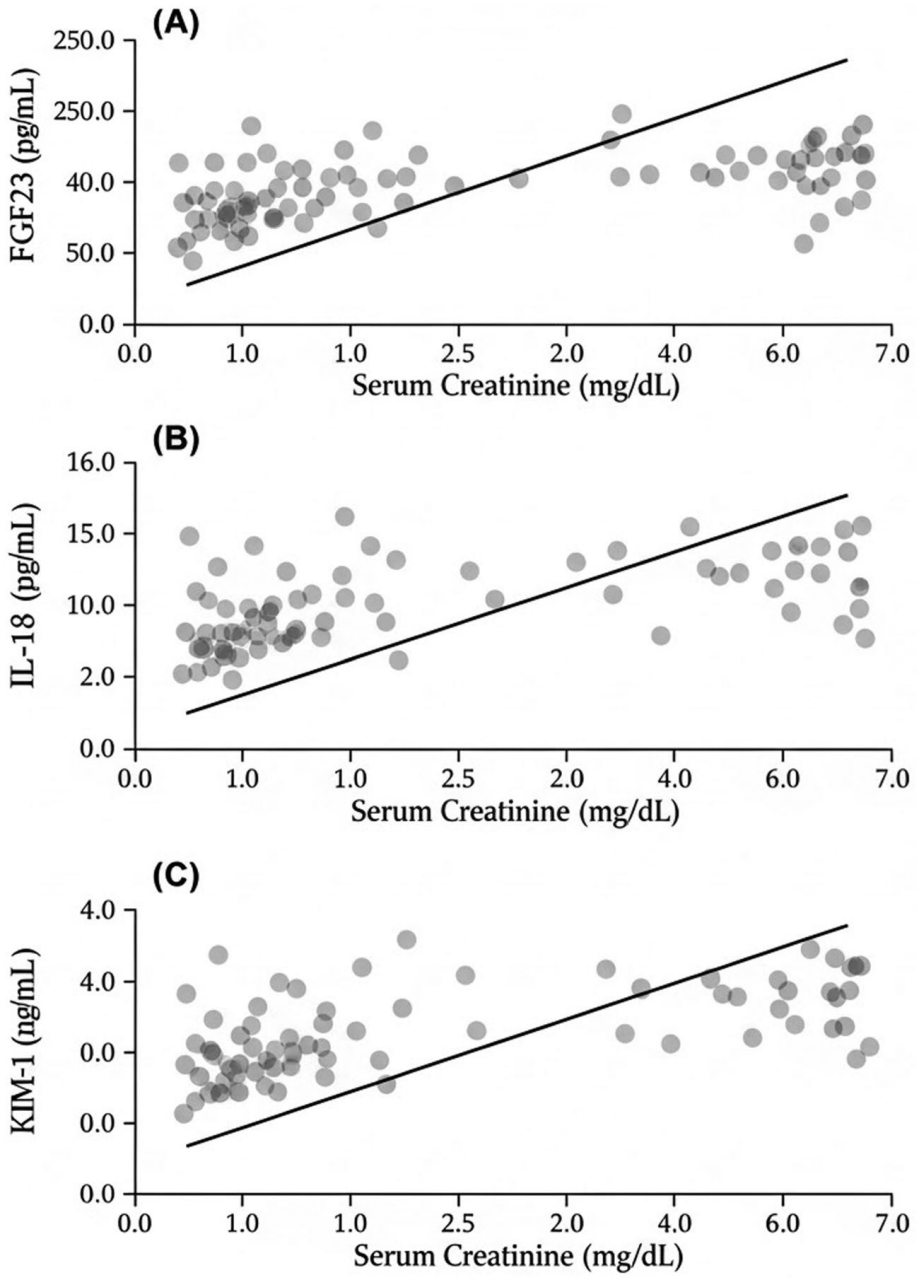
Association between post-paracentesis biomarker levels and peak serum creatinine within 72 h. Scatter plots demonstrating the correlation between post-paracentesis biomarker concentrations and peak serum creatinine measured within 72 h after paracentesis. (A) FGF-23; (B) IL-18; (C) KIM-1. Each point represents an individual patient. The solid line represents the linear regression fit. Pearson correlation coefficients were as follows: IL-18 (*r* = .632, *p* <.001), KIM-1 (*r* = .556, *p* < .001), and FGF-23 (*r* = .687, *p* < .001). IL-18: interleukin-18; KIM-1: kidney injury molecule-1; FGF-23: fibroblast growth factor-23.

Prices of post-paracentesis biomarkers were positively correlated with the highest concentrations of serum creatinine at 72 h post-procedure. The correlation coefficient was highest with FGF-23 (*r* = .687, *p* < .001) then followed by IL-18 (*r* = .632, *p* < .001) and KIM-1 (*r* = .556, *p* < .001) (Figure 2).

Post-paracentesis IL-18, KIM-1 and FGF-23 were significantly related with AKI (all *p* < .001), but not MELD score, baseline serum creatinine, CRP and paracentesis volume. An MELD score, baseline serum creatinine, and post-paracentesis FGF-23 multivariate logistic regression model showed that FGF-23 was independently related to AKI (adjusted odds ratio 1.08 per 10 pg/mL change; 95% CI 1.031.14; *p* < .001). In the adjusted model, no independent association of AKI was found with MELD score and baseline creatinine (Table 3). The last model was well calibrated (Hosmer–Lemeshow *p* = .62) and high discriminating (C-statistic 0.91). The association of IL-18 or KIM-1 with a comparable adjusted model was reduced when FGF-23 was added.

**Table 3.** Multivariable logistic regression analysis for predictors of post-paracentesis AKI.

Variable	Adjusted OR	95% CI	*p* Value
MELD score (per 1-point increase)	1.04	0.98–1.11	.18
Baseline creatinine (per 0.1 mg/dL increase)	1.72	0.89–3.31	.10
FGF-23 (per 10 pg/mL increase)	1.08	1.03–1.14	<.001

*Note:* Model performance: Hosmer–Lemeshow *p* = .62; C-statistic = 0.91. OR: odds ratio; CI: confidence interval; MELD: Model for End-Stage Liver Disease; FGF-23: fibroblast growth factor-23.

## Discussion

In this prospective cohort of decompensated cirrhosis receiving therapeutic paracentesis, we found that early increase in IL-18, KIM-1, and FGF-23 were highly related to AKI in the post-procedural period and baseline was similar in those who developed and did not develop AKI. This trend can help to justify the idea that these biomarkers reflect acute kidney stress caused by a paracentesis and not primary structural renal disease. Recent information in cirrhosis and critical disease demonstrate that tubular and inflammatory biomarkers frequently increase prior to creatinine, and they can enhance stratify AKI hazards and forecast similarly to traditional markers. It is worth noting that FGF 23 was most associated with peak creatinine, highest ROC discrimination and still independently correlated with AKI in multivariate models, which is consistent with current evidence that FGF 23 is a powerful early nephrology predictor and response in a wide range of high-risk groups [2,15–21].

Clinical significance of such a deficit of baseline difference between groups is apparent in cirrhosis, where creatinine is a late and insensitive marker of renal injury due to sarcopenia, abnormal hepatic creatine countenance, distended extra-cellular volume, and tubular hypersecretion. The pre-creatinine-based AKI criteria increase in IL 18, KIM-1 and FGF 23 during the 24 h after paracentesis-prior to the onset of creatinine-based AKI criteria, is supportive of a mechanistic cascade where the effective arterial underfilling and the occurring instability of paracentesis-induced circulatory dysfunction results in early tubular ischemia and inflammatory stress. IL-18 and KIM-1 have been determined to be markers of ischemic and inflammatory tubular injury in cirrhosis and decompensated chronic liver disease and are used to distinguish between structural AKI phenotypes (acute tubular necrosis and hepatorenal syndrome) and functional prerenal conditions. Conversely, FGF 23, a phosphaturic hormone synthesized mainly by osteocytes, exhibits pleiotropic actions on inflammation and endothelial action and mineral metabolism; its concentrations rise quickly in AKI, possibly due to a combination of increased secretion and decreased renal clearance, and inflammatory control. This wider biologic footprint could be the reason, why FGF 23 was the best risk capture in our cohort compared to IL-18 or KIM −1 [2,15,16,19,20,22].

We find that FGF 23 gave the strongest AUC and was the only independent variable linked to AKI after controlling the variable of MELD and baseline creatinine, which is in line with recent findings in critically ill patients, postoperative groups and general AKI patients, where intact and C-terminal FGF 23 are more closely associated with AKI onset, severity, and outcome than are conventional markers. In these researches, KIM-1 is also likely to be more strongly associated with initial tubular epithelial damage and AKI development, whereas FGF-23 follows the severity and subsequent complications, which could indicate partially complementary functions. Univariable but weaker multivariate IL-18 and KIM-1 in our cirrhotic cohort may indicate overlapping pathways of inflammation and injury that are more comprehensively reflected by FGF-23, and also the hemodynamic and inflammatory environment unique to the decompensated cirrhosis [2,15–18,20].

The information is also combined with the accumulating knowledge of new AKI biomarkers in cirrhosis, and in the specific case of paracentesis. Recent studies in decompensated cirrhosis receiving moderate-volume paracentesis established that urinary TIMP 2-IGFBP7 increases promptly and can predict rapid GFR fall, hemodynamic incidents, and major adverse kidney events, supporting the idea that urinary TIMP 2-IGFBP7 is a rapid responder to the circulatory effects of paracentesis. Huge cohorts with cirrhosis-specific studies have shown that NGAL, IL-18, and KIM-1 do not only differentiate structural and functional AKI but also predict progression and short-term mortality and can be used to refine treatment decisions as terlipressin use in hepatorenal syndrome. It is within this context that our results help to augment the biomarker framework by suggesting that incremental prognostic information can be offered by FGF-23, which has received enormous research in general AKI, in the post-paracentesis era compared to the use of tubular and inflammatory biomarkers alone [2,15,20].

Clinically, the observed high sensitivity and specificity of the identified FGF 23 cutoff allow considering integrating early biomarker evaluation with the routine post-paracentesis care packages. The same 24 h post-procedure, an increased FGF 23 perhaps combined with IL-18, KIM-1, or other tubular indicators may be able to detect a subset of patients at high risk of AKI who could be benefited by: (1) more vigilant hemodynamic and urine output monitoring; (2) more vigorous or protracted albumin support; (3) earlier attention to vasoconstrictor therapy in the patients who have features suggestive of an evolving hepatorenal syndrome; and (4) strict avoidance of ne. These approaches are consistent with modern suggestions of biomarker-based AKI prevention, where a biomarker increase causes a programmed biomarker-mediated renal protection reaction prior to the onset of functional impairment. Notably, FGF-23 and similar biomarkers play their role in this situation as an early warning mechanism that supplements, but does not substitute, clinical judgment and standard parameters [15–17,23].

Our study has various limitations that should be highlighted. Single-center design and the small sample size could affect the overall validity of the results, especially when it comes to various etiologies of cirrhosis, various paracentesis volumes, and different approaches to albumin replacement. Biomarker measurements were not done after 24 h, which excludes any determination of biomarker patterns, how they relate to renal recovery versus chronic AKI and whether they can be used to guide de-escalation in response to falling levels. We also failed to determine more specific hemodynamic indices, renin 5 angiotensin5 Aldosterone activation, or cytokine profiles, which would help illuminate mechanistic relationships between circulatory dysfunction caused by paracentesis, systemic inflammation, and FGF 23 secretion. Just like in the majority of observational biomarker studies, residual confounding could still exist in spite of multivariate adjustment. Finally, the viability of the considerations, such as assay standardization (intact vs C-terminal FGF-23), cost, turnaround time, and availability, can be limiting to the immediate implementation, specifically in those resource-limited settings with high cirrhosis burden [18,19].

Future studies should take a number of paths. First, multicenter validation in larger cohorts with different paracentesis practices is required to confirm the performance characteristics of FGF-23, IL-18, and KIM-1 and define clinically actionable thresholds. Second, longitudinal designs with dense sampling could characterize biomarker kinetics, distinguish patterns between transient hemodynamic perturbation and evolving structural AKI, and assess the incremental value of combining FGF-23 with tubular injury or cell-cycle arrest markers such as NGAL and TIMP-2·IGFBP7. Third, mechanistic studies are required to understand how FGF-23 interacts with endothelial dysfunction, oxidative stress, and inflammatory signaling in the cirrhotic liver and kidney based on experimental data linking FGF-23/klotho pathways to vascular injury, fibrosis, and repair in AKI models. Finally, interventional trials should test biomarker-guided strategies such as tailored albumin dosing or early vasoconstrictor initiation to see if integrating FGF-23 and tubular biomarkers into post-paracentesis protocols can reduce AKI incidence or improve survival in this vulnerable population [18,19,23].

In conclusion, our data show that early post-paracentesis elevations in IL-18, KIM-1, and especially FGF-23 are strongly and independently associated with subsequent AKI in decompensated cirrhosis. Consistent with evolving evidence positioning FGF-23 as a dynamic biomarker of AKI onset and severity, FGF-23 in this cohort has shown superior correlation with renal dysfunction as well as excellent discriminatory performance plus predictive value beyond conventional clinical variables and tubular injury markers. These findings support further evaluation of FGF-23 alone and in multimarker panels as an early risk stratification tool to guide preventive strategies after therapeutic paracentesis in high-risk cirrhotic patients.

## Conclusion

IL-18 increase, KIM-1 and in particular FGF-23, early after paracentesis were highly predictive for AKI in patients with decompensated cirrhosis. FGF-23 was the most discriminating and retained independent association with AKI even after receiving baseline renal function and the severity of liver disease. These novel observations justify further validation of FGF-23 as an early risk stratification marker prior to clinical introduction.

## Data Availability

This content has not been published before, in whole or in part, and is not being considered for publication elsewhere. This work contains no tables or figures that require permission to republish. The datasets used and analyzed during the current study are available from the corresponding author on reasonable request.
